# Comparative analysis of machine learning techniques for cardiovascular disease prediction

**DOI:** 10.1371/journal.pone.0356170

**Published:** 2026-08-18

**Authors:** Md. Mahfuz Uddin, Md. Binyamin, Md. Muhtasim Munif Fahim, Md. Rezaul Karim

**Affiliations:** 1 Data Science Research Lab, Department of Statistics, University of Rajshahi, Rajshahi, Bangladesh; 2 Department of Statistics, Mawlana Bhashani Science and Technology University, Tangail, Bangladesh; The University of Aukland, NEW ZEALAND

## Abstract

**Background and objectives:**

Early and accurate prediction of cardiovascular disease (CVD) is fundamental for reducing morbidity and mortality. Machine learning (ML) algorithms provide a data-driven, actionable foundation to strengthen clinical decision-making and enable more precise risk stratification. The purpose of this study is to identify the most significant risk factors for CVD and to compare the predictive performance of eight machine learning algorithms.

**Materials and methods:**

The study utilizes the Cardiovascular Disease dataset, an open-access resource from the Kaggle repository. It applies 5-, 10-, 15-, and 20-fold cross-validation (CV) to evaluate the performance of Logistic Regression, Decision Tree, Random Forest, Support Vector Machine (SVM), XGBoost, LogitBoost, Gradient Boosting, and LightGBM. Accuracy, sensitivity, specificity, precision, F1-score, false discovery rate (FDR), and area under the receiver operating characteristic curve (AUC) are used to evaluate the performance of the algorithms. The selection and ranking of relevant features are achieved through multiple methodologies, including Boruta, Regularized Random Forest, Recursive Feature Elimination, and LASSO.

**Results:**

All clinical and demographic characteristics except gender show significant differences between the CVD and non-CVD groups (p-values <0.001). While 5-fold cross-validation yields somewhat better metrics for most techniques, model performance remains stable across folds. With the best AUC of 0.800 (95% CI: 0.792–0.807) and balanced F1-score(0.77), LightGBM produces the most balanced findings while retaining a high sensitivity (0.791) under both 5-fold and 20-fold CV. Despite having low sensitivity (0.628), LogitBoost achieves the best accuracy (0.814) and specificity (0.911) in 20-fold CV. Considering all evaluation metrics, particularly AUC and sensitivity, LightGBM with 5-fold cross-validation is selected as the optimal model for CVD prediction. Gain analysis indicates that systolic blood pressure is the primary contributor, and feature selection techniques repeatedly identified systolic blood pressure, age, and cholesterol levels as prominent predictors.

**Conclusion:**

LightGBM demonstrates modestly superior, more balanced performance compared to the other evaluated algorithms in this dataset. While the performance (AUC = 0.800) suggests potential utility as a decision support tool, external validation is needed before clinical deployment. The model is not yet ready for standalone clinical use.

## 1. Introduction

Cardiovascular disease (CVD) is a broad term that refers to a group of disorders affecting the heart and blood vessels, and it is the common cause of heart failure worldwide [1]. The term “CVDs” refers to a collection of illnesses that affect the heart and blood vessels, such as rheumatic heart disease, coronary heart disease, and cerebrovascular disease. An estimated 17.9 million people worldwide died from cardiovascular diseases (CVDs), making them the top cause of death worldwide. Heart attacks and strokes account for around four out of every five deaths from CVD, with one-third of these deaths occurring in those under the age of 70 [2]. Several contributing risk factors, including diabetes, high blood pressure, excessive cholesterol, irregular pulse rate, and many more, make it challenging to diagnose heart disease. The European Cardiology Society (ESC) reports that 26 million adults worldwide have been diagnosed with heart disease, and 3.6 million more are diagnosed with the condition each year. Nearly half of all patients with heart disease died within a year or two, and treating the condition costs close to 3% of the total health care expenditure [3]. Numerous data mining and neural network techniques have been used to assess the severity of heart disease in humans [4]. The last 50 years have witnessed unprecedented advances in CVD, as evidenced by the notable and continuous decline in CVD-related mortality. Nevertheless, despite these advancements, CVD continues to be the largest cause of death and disability in the US and around the world, impacting 85.6 million people and spending one out of every six healthcare dollars. Recent trends are alarming, even as they offer promise for new treatment models and further opportunities for biomedical innovation [5].

In recent years, machine learning (ML) has proven to be an effective tool for revealing hidden patterns in big, complex datasets and improving predictive accuracy. To provide individualized risk forecasts and early diagnosis, machine learning techniques can integrate a variety of organized and unstructured data, including genetic markers, imaging, clinical measurements, and electronic health records. A study [6] was conducted using the “Framingham” dataset, obtained from Kaggle for analysis. The UCI Machine Learning Repository provided a dataset of 14 features related to heart disease. The study evaluated 14 essential features for prediction, whereas previous surveys included only 10. Furthermore, the article compared machine learning techniques, including Random Forest (RF), Logistic Regression, Support Vector Machine (SVM), and Naïve Bayes, for the classification of cardiovascular diseases. Based on a comparison, the Random Forest machine learning algorithm was used in the study because it was the most reliable and accurate.

The “Heart Disease Dataset” from the UCI Machine Learning Repository included 14 characteristics that could be used to determine whether heart disease was present. Five machine learning techniques, XGBoost (Extreme Gradient Boosting), Random Forest, Decision Tree, Support Vector Machine, and k-Nearest Neighbor were used in [7]. The study suggested that feature selection and ensemble methods, such as stacking, improved the predictive accuracy of weak classifiers and performed satisfactorily in identifying the risk of heart disease [7]. Nine machine learning techniques, including K-Nearest Neighbors, Support Vector Machine, Decision Tree, Artificial Neural Network, Naïve Bayes, Random Forest, Logistic Regression, Voting Classifier, and Gradient Boosting, were applied to the Cardiovascular Disease Dataset available on Kaggle [8]. On the dataset, the Gradient Boosting model performed better than the others, achieving 73% accuracy, 73% recall, 73% F1-score, and 74% precision [8]. A machine learning model for cardiovascular disease risk prediction was developed in [9] using a dataset with 11 variables. The results were derived from the approximately 70,000 patient records in the Kaggle dataset on cardiovascular illness. For this dataset, models developed with C5.0, QUEST, Bayesian networks, random forests, and neural networks were compared. On the training and test data sets, a high accuracy of 99.1% was achieved, which was notably superior to previous methods. The suggested prediction model was expected to lead to better treatment results as well as early detection and diagnosis of cardiac disease.

To assess how well machine learning algorithms predict myocardial infarction, several clinical characteristics were collected from heart failure patients [10]. Six machine learning models, including Logistic Regression, Support Vector Machine, XGBoost, LightGBM, Decision Tree, and Bagging, were evaluated using key performance metrics such as accuracy, precision, recall, F1 Score, and AUC. The paper showed that XGBoost performed best, achieving 94.80% accuracy and an AUC of 90.0%. LightGBM placed second with an accuracy of 92.50% and an AUC of 92.00%. It was demonstrated that logistic regression was a reliable option, with an accuracy of 85.0% [10]. The study highlights how machine learning can improve the prognosis of myocardial infarction and provide insights for clinical judgment and healthcare intervention strategies. The study shows how machine learning could help with myocardial infarction prognosis and offer useful data for clinical decision-making and healthcare intervention strategies. The paper [11] applied machine learning methods for the early detection of heart conditions, especially myocardial infarction. The work provided valuable insights for developing trustworthy myocardial infarction prediction models by analyzing the performance of multiple classifiers. Its findings demonstrated the potential to fine-tune an XGBoost model for cardiovascular conditions, achieving 98.71% F1 score, 98.29% recall, 99.14% precision, and 98.50% accuracy.

One of the most important steps in improving the prediction accuracy and interpretability of machine learning models is selecting suitable features from a dataset. Even though several studies using machine learning approaches to predict cardiovascular disease (CVD) have been published, several methodological issues remain. It is challenging to evaluate the stability and consistency of predictor relevance across approaches because most current studies rely on only a few feature selection techniques. In the current CVD literature, advanced feature selection methods such as the Boruta algorithm, Regularized Random Forest (RRF), Recursive Feature Elimination (RFE), and Least Absolute Shrinkage and Selection Operator (LASSO) have been somewhat neglected. In the previous research, powerful ensemble-based machine learning algorithms, such as Extreme Gradient Boosting (XGBoost), LogitBoost, and LightGBM, have not been thoroughly assessed alongside conventional approaches. Additionally, the clinical transparency of machine learning-based predictions has been limited by the infrequent use of interpretability methods to analyze the contributions and influences of predictors in top-performing models.

To address these gaps, the current study uses a publicly available cardiovascular disease dataset from Kaggle to perform a thorough comparative analysis of eight machine learning algorithms: Logistic Regression, Decision Tree, Random Forest, Support Vector Machine (SVM), Extreme Gradient Boosting (XGBoost), LogitBoost, Gradient Boosting, and LightGBM. Key performance indicators, such as accuracy, sensitivity, specificity, precision, F1-score, and area under the receiver operating characteristic curve (AUC) are used in the study to assess the model’s performance. To find the most significant predictors of CVD, four sophisticated feature selection techniques, such as Boruta, RRF, RFE, and LASSO, are used in addition to model comparison. Additionally, to enhance interpretability and offer clinically significant insights into cardiovascular risk prediction, the study integrates SHAP-based feature importance and contribution analysis for the top-performing model. Thus, this study adds incremental methodological knowledge about feature stability, effective model validation, and interpretable machine learning for early CVD diagnosis and intervention, in addition to determining the optimal predictive model. That is, the study provides modest incremental contributions to the cardiovascular disease prediction literature, primarily in algorithmic feature engineering and model validation, rather than in cardiovascular pathology.

**Fig 1:**
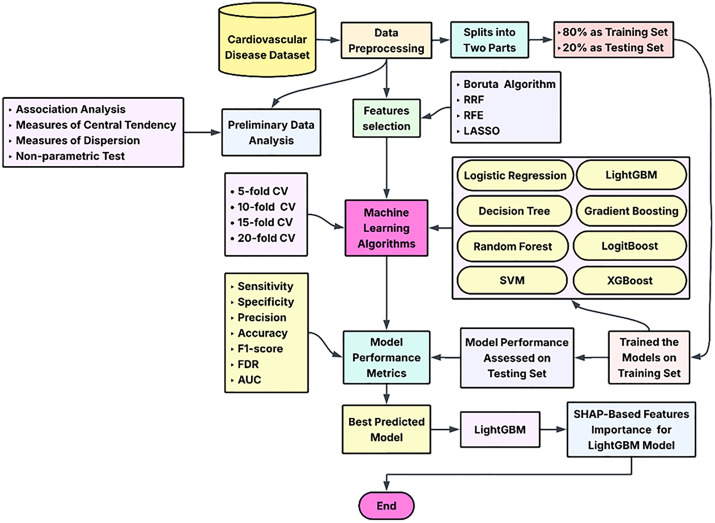
Proposed study diagram.

Fig 1 presents a diagram of the proposed study.

The remaining part of the manuscript is structured as follows: The Materials and Methods section presents the materials and methods used in this study. The Results section describes the results, and the Discussion section provides a comprehensive discussion of the findings. Finally, the Conclusion and Future Research section concludes and suggests a few directions for future research.

## 2. Materials and methods

### 2.1. Data source and study variables

The dataset used in this study includes 68205 observations for 17 variables and is obtained from the publicly accessible Kaggle database. The main goal is to use a variety of patient parameters to predict whether cardiovascular disease will be present [12]. The detailed description of the dataset is available at: https://www.kaggle.com/datasets/colewelkins/cardiovascular-disease.

The research does not require an ethics statement because we analyzed a publicly available secondary dataset with no direct contact with human subjects.

### 2.2. Data preprocessing

The dataset initially contains 6,494 outliers, which are identified using the Interquartile Range (IQR) method, defined as values below ${\text{Q}}_{\text{1}}-\;\text{1}.\text{5}\times \text{IQR}$ or above ${\text{Q}}_{\text{3}}\;+\;\text{1}.\text{5}\times \text{IQR}$. BMI has the most outliers (1,988), followed by height (502), age in days (4), age in years (4), weight (1,711), diastolic blood pressure (1,637), and systolic blood pressure (648). These outliers are subsequently removed to improve data quality. In addition, six variables are excluded during preprocessing to reduce redundancy and improve model interpretability. Specifically, the variable ‘id’ is removed because it serves as a unique patient identifier with no predictive relevance. ‘Age (in days)’ is converted into age (years) to improve interpretability and only age (years) is retained in the final analysis. We also remove ‘BP category’ as it is derived from systolic and diastolic BP following ESC guidelines, while ‘BP category encoded’ is excluded as it is derived directly from the BP category variable and can introduce redundancy. Furthermore, ‘Height’ and ‘weight’ are also excluded because BMI, calculated as weight/height², is retained as a more informative composite measure of body composition. After preprocessing, the final dataset consists of 61,711 observations and 11 variables, including 10 explanatory variables and 1 response variable. The explanatory variables are gender (2 categories), age(years), systolic BP, diastolic BP, cholesterol levels (3 categories), glucose levels (3 categories), smoking status (2 categories), alcohol intake (2 categories), physical activity (2 categories), and BMI. The response variable is ‘cardio’ (2 categories). All subsequent analyses are performed based on this refined dataset.

### 2.3. Statistical analysis

To explore the characteristics of the variables, various descriptive and inferential statistical techniques are employed, including frequency analysis, measures of central tendency and dispersion, skewness and kurtosis, the one-sample Kolmogorov-Smirnov (K-S) test, and the nonparametric Mann-Whitney U test.

### 2.4. Features selection

Several types of feature selection techniques, including Boruta algorithm [13,14], Regularized Random Forest (RRF) [15,16], Recursive Feature Elimination (RFE) [17,18], and LASSO [19,20] are applied for selecting the most important predictors for the prediction of cardiovascular disease.

### 2.5. Machine learning algorithms

Eight machine learning algorithms, including Logistic Regression [21,22], Decision Tree [23,24], Random Forest [25–27], Support Vector Machine(SVM) [28,29], XGBoost [30,31], LogitBoost [32,33], Gradient Boosting [31,34], and LightGBM [35,36] have been utilized for predicting cardiovascular disease. To ensure accuracy and reliability, the models are trained and evaluated using appropriate performance criteria.

### 2.6. Model evaluation metrics

The dataset is initially divided into training (80%, N = 49,370) and a test (20%, N = 12,341) sets using stratified sampling to preserve the distribution of CVD status. For model training, validation, and hyperparameter tuning, cross-validation (5-, 10-, 15-, and 20-fold) is applied only to the training set. The held-out test set is used to evaluate the final model’s performance, including ROC curves and evaluation metrics, to ensure an objective assessment of predictive performance. The k-fold cross-validation with k > 10 can lead to highly overlapping training sets and increased evaluation variance; we included 15- and 20-fold CV primarily to demonstrate the stability of the results. To evaluate the performance of the machine learning models, this study employs the evaluation metrics of Table 1.

**Table 1:** Performance evaluation metrics used for model assessment.

Metrics	Formula	Description
Sensitivity	$\frac{\text{TP}}{\text{TP}+\text{FN}}$	Determines the proportion of true positives accurately identified.
Specificity	$\frac{\text{TN}}{\text{FP}+\text{TN}}$	Determines the proportion of true negatives accurately identified.
Precision	$\frac{\text{TP}}{\text{TP}+\text{FP}}$	Measures how many predicted positives are actually correct.
Accuracy	$\frac{\text{TP}+\text{TN}}{\text{TP}+\text{TN }+\text{FP}+\text{FN}}$	Calculates the overall model accuracy.
F1-score	$\frac{\text{2TP}}{\text{2TP}+\text{FP}+\text{FN}}$	Balancing both the harmonic mean of sensitivity and precision.
FDR	$\frac{\text{FP}}{\text{TP}+\text{FP}}$	Determines the proportion of false positives among predicted positives.

Here TP = True Positive, FN = False Negative, FP = False Positive, TN = True Negative [37, 38].

### 2.7. Hyperparamter tuning

Hyperparameter tuning is the process of identifying the optimal values of model hyperparameters to achieve the best predictive performance. Hyperparameters are predefined settings that control the learning process of a machine learning algorithm and are not estimated directly from the training data. These regulate several aspects of the learning process and are usually established before the start of the training session. Effective tuning improves pattern recognition, prevents overfitting and underfitting, and increases accuracy on unseen data. Details can be found in [39,40].

### 2.8. SHAP-based features importance method

A popular method for evaluating machine learning model predictions is Shapley Additive exPlanation (SHAP). SHAP provides information about how each attribute contributes to certain predictions using Game Theory techniques. It is a member of a class of additive feature attributions, model-agnostic techniques that work with a variety of machine learning and deep learning models. These techniques improve understanding of model behavior by giving individual input attributes significance [41]. By calculating Shapley values derived from cooperative game theory, it provides consistent, locally accurate feature attributions and enables both local interpretation to explain individual predictions and global interpretation to highlight the most influential characteristics across the dataset [42]. A detailed explanation can be found in [43,44].

## 3. Results

### 3.1. Descriptive statistics

The descriptive statistics provided in Table 2 represent key insights into the dataset. Age (years) ranges from 39 to 64, with a mean of 52.89 years and a coefficient of variation (CV) of 12.75%, indicating moderate variability. BMI shows a moderate dispersion (CV = 15.94%) and an average of 26.94. With a mean of 126.30 mmHg, systolic blood pressure (mmHg) shows greater skewness (0.74), suggesting the presence of extreme values. There is minor skewness (0.45), although the diastolic blood pressure (mmHg) is better centered (mean = 81.65 mmHg). The distribution of the variables indicates considerable asymmetry, especially in BMI and systolic blood pressure, which may signal underlying health inequalities in the community.

**Table 2:** Descriptive statistics of quantitative variables.

Statistics	Systolic BP (mmHg)	Diastolic BP (mmHg)	Age (years)	BMI
Minimum	90.00	65.00	39.00	14.53
Maximum	170.00	105.00	64.00	39.48
1^st^ quartile	120.00	80.00	48.00	23.85
Median	120.0	80.00	54.00	26.17
Mean	126.30	81.65	52.89	26.94
3^rd^ quartile	140.00	90.00	58.00	29.69
SD	14.25	7.65	6.75	4.29
CV	11.28	9.37	12.75	15.94
Skewness	0.74	0.45	−0.31	0.57
Kurtosis	0.28	0.08	−0.81	−0.09

### 3.2. Non-parametric test

Mann-Whitney U test results in Table 3 show that the CVD and non-CVD groups differ statistically significantly on all quantitative measures (p-value < 0.001). Systolic blood pressure (r = 0.440) and diastolic blood pressure (r = 0.338) show moderate effects based on effect size analysis, suggesting comparatively stronger associations with CVD status. However, despite their statistical significance, age (years) (r = 0.235) and BMI (r = 0.175) display minor effect sizes, indicating more moderate group differences.

**Table 3:** Mann-Whitney U (Wilcoxon Rank-Sum) test for normality for quantitative variables.

Variables	Test statistic (W-value)	P-value	Effect size (r)	95% CI	Magnitude
Age (years)	347059036	<0.001	0.235	0.230-0.240	small
Systolic BP	244568001	<0.001	0.440	0.430-0.450	moderate
Diastolic BP	307148465	<0.001	0.338	0.330-0.340	moderate
BMI	379752459	<0.001	0.175	0.170-0.180	small

### 3.3. Association analysis

Table 4 compares demographic, clinical, and lifestyle variables between participants with and without CVD. The final preprocessed dataset comprises 61,711 observations, of which 30,318 (49.13%) are classified as CVD-positive and 31,393 (50.87%) as CVD-negative, indicating a well-balanced class distribution (ratio approximately 1:1.04). All quantitative variables differ significantly between the two groups (p-value < 0.001). Individuals with CVD are older (54.51 vs. 51.32 years), have systolic BP (132.60 vs. 120.30 mmHg), diastolic BP (84.26 vs. 79.12 mmHg), and BMI (27.70 vs. 26.20 kg/m²). Among categorical variables, gender distribution does not differ significantly (p-value = 0.776). However, participants with CVD are more likely to have elevated cholesterol and glucose levels. The prevalence of “Well Above Normal” cholesterol is notably higher in the CVD group (17.22% vs. 5.29%). The CVD group also has slightly higher rates of non-smoking (91.88% vs. 90.70%) and non-alcohol consumption (95.05% vs. 94.50%), but a greater proportion are physically inactive (21.01% vs. 18.11%, p-value < 0.001).

**Table 4:** Association test between dependent and independent variables.

Covariates	Cardiovascular disease		
	No (N = 31393)	Yes (N = 30318)	P-value
	Mean± SD	Mean± SD	
**Age (years)**	51.32±6.75	54.51±6.34	<0.001
**Systolic BP**	120.30±10.63	132.60±14.80	<0.001
**Diastolic BP**	79.12±6.44	84.26±7.93	<0.001
**BMI**	26.20±4.08	27.70±4.37	<0.001
**Gender**	N(%)	N(%)	
Female	20300 (64.66)	19639 (64.78)	0.776
Male	11093 (35.33)	10679 (35.22)	
**Cholesterol levels**			
Normal	26448 (84.25)	20308 (66.99)	<0.001
Above Normal	3285 (10.46)	4788 (15.79)	
Well Above Normal	1660 (5.29)	5222 (17.22)	
**Glucose levels**			
Normal	27792 (88.53)	25010 (82.49)	<0.001
Above Normal	1821 (5.80)	2484 (8.19)	
Well Above Normal	1780 (5.67)	2824 (9.32)	
**Smoking status**			
Non-smoker	28472 (90.70)	27857 (91.88)	<0.001
Smoker	2921 (9.30)	2461 (8.12)	
**Alcohol intake**			
Does not consume alcohol	29667 (94.50)	28816 (95.05)	<0.003
Consumes alcohol	1726 (5.50)	1502 (4.95)	
**Physical activity**			
Not physically active	5684 (18.11)	6371 (21.01)	<0.001
Physically active	25709 (81.89)	23947 (78.99)	

For quantitative variables, the p-values are derived from the Mann-Whitney U test statistic, while for categorical variables, the p-values are obtained from the Chi-square test statistic.

### 3.4. Feature selection

**Fig 2:**
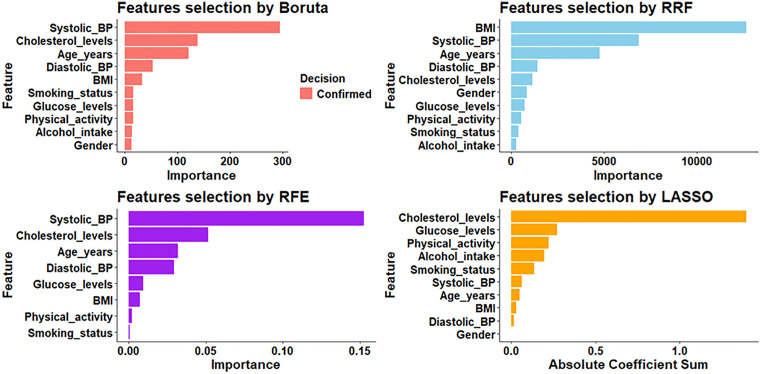
Feature selection based on the whole dataset.

Feature selection is performed on the entire dataset prior to model development to identify a globally consistent set of predictive variables. Fig 2 illustrates the feature selection results for the whole dataset obtained using four advanced feature selection techniques: Boruta algorithm, Regularized Random Forest (RRF), Recursive Feature Elimination (RFE), and LASSO. Systolic blood pressure consistently demonstrates substantial discriminative potential as the most significant predictor of cardiovascular illness (CVD) across Boruta, RRF, and RFE. Frequently rated among the top features are other variables, including age, BMI, and cholesterol levels, indicating their importance in predicting cardiovascular outcomes. The LASSO approach, which penalizes less informative variables by reducing their coefficients, identifies cholesterol levels and glucose levels as the most important predictors.. Physical activity, smoking status, and alcohol consumption are behavioral characteristics that are thought to have less of an impact across most methodologies, particularly in RFE and RRF. In predicting cardiovascular illness, all 10 characteristics have some impact, though the importance levels differ across techniques. Even lower-ranked characteristics like physical activity, alcohol consumption, and smoking status are kept by at least one selection strategy, even if variables like age, systolic blood pressure, cholesterol levels, and BMI regularly score highly. Thus, each variable has predictive value, albeit to varying degrees, and when taken into account as a whole, could improve model performance.

### 3.5. Hyperparameter tuning

Hyperparameter tuning is performed to optimize the performance of all machine learning models shown in Table 5. Different combinations of model-specific hyperparameters are carefully evaluated using a grid-search technique and 5-fold cross-validation. The best cross-validation performance (highest accuracy or minimal log-loss, depending on the technique) is used to determine the ideal hyperparameter set for each model. This process reduces the risk of overfitting and improves model generalization.

**Table 5:** Optimal hyperparameter settings of machine learning models obtained using grid search with 5-fold cross-validation.

Model names	Best Hyperparameters
Logistic Regression	alpha = 1, lambda = 0.0001
Decision Tree	cp = 0.0002, max_depth = 20, min_split = 20
Random Forest	mtry = 2, ntree = 500
SVM	sigma = 0.176, C = 1
XGBoost	nrounds = 526, max_depth = 3, eta = 0.125, gamma = 5.776, colsample_bytree = 0.550, min_child_weight = 21, subsample = 0.781
LogitBoost	nIter = 50
Gradient Boosting	n.trees = 25, interaction.depth = 4, shrinkage = 0.325, n.minobsinnode = 5
LightGBM	learning_rate = 0.05, num_leaves = 31, max_depth = 5, nrounds = 300, best_iter = 40, best_score = 0.546

### 3.6. Evaluation of model performance

Table 6 presents the performance of eight machine learning algorithms evaluated using 5-, 10-, 15-, and 20-fold cross-validation. For model training and hyperparameter tuning, cross-validation procedures (5-, 10-, 15-, and 20-fold) are applied to the training set. The independent held-out test set is then used to analyze every performance metric listed in Table 6. From the analysis, we observe that the performance metrics are relatively stable across different cross-validation schemes, indicating the robustness of the models. Among all models, LightGBM achieves the highest discriminative ability, with an AUC of 0.800 (95% CI: 0.792–0.807) under both 5-fold and 20-fold cross-validation. LightGBM also demonstrates balanced sensitivity (0.791), specificity (0.661), precision (0.707), and F1-score (0.747), suggesting strong overall classification performance. Random Forest performs comparably, with an AUC of 0.799 and the highest sensitivity (0.796) under 10-fold cross-validation. XGBoost and Gradient Boosting also show strong performance, with AUC values of 0.784–0.787, while Logistic Regression, SVM, and Decision Tree achieve moderate discrimination. LogitBoost has the highest accuracy (0.814) and specificity (0.911) under 20-fold cross-validation, but its sensitivity (0.628) and AUC (0.715) are lower than those of other models. Considering all evaluation metrics, particularly AUC and sensitivity, LightGBM with 5-fold cross-validation is selected as the optimal model for CVD prediction. The model provides the best overall discrimination while maintaining a balanced trade-off between correctly identifying diseased and non-diseased individuals.

**Table 6:** Evaluation of model performance metrics for eight ML algorithms using 5-, 10-, 15-, and 20-fold CV.

Model	CV (folds)	Performance metrics						
		Sensi tivity	Specificity	Precision	Accuracy	F1- score	FDR	AUC (95% CI)
Logistic Regression	5	0.787	0.653	0.701	0.721	0.742	0.298	0.777 (0.769-0.785)
	10	0.791	0.651	0.701	0.722	0.744	0.299	0.776 (0.768-0.785)
	15	0.788	0.653	0.702	0.721	0.742	0.298	0.777 (0.769-0.785)
	20	0.788	0.653	0.702	0.722	0.742	0.298	0.777 (0.770-0.785)
Decision Tree	5	0.783	0.676	0.715	0.731	0.747	0.285	0.783 (0.776-0.802)
	10	0.780	0.674	0.712	0.728	0.745	0.288	0.786 (0.778-0.795)
	15	0.780	0.674	0.712	0.728	0.745	0.288	0.786 (0.778-0.795)
	20	0.783	0.676	0.715	0.731	0.747	0.285	0.783 (0.776-0.803)
Random Forest	5	0.795	0.656	0.705	0.727	0.748	0.295	0.798 (0.790-0.806)
	10	0.796	0.653	0.704	0.726	0.747	0.296	0.799 (0.791-0.807)
	15	0.791	0.660	0.707	0.727	0.746	0.293	0.798 (0.790-806)
	20	0.793	0.658	0.706	0.727	0.747	0.294	0.799 (790-0.807)
SVM	5	0.781	0.665	0.707	0.724	0.742	0.293	0.762 (0.753-0.770)
	10	0.782	0.665	0.707	0.724	0.742	0.293	0.762 (0.753-0.770)
	15	0.782	0.664	0.707	0.724	0.742	0.293	0.762 (0.754-0.770)
	20	0.783	0.663	0.707	0.724	0.743	0.293	0.762 (0.754-0.770)
XGBoost	5	0.786	0.673	0.713	0.730	0.748	0.286	0.787 (0.779 −0.795)
	10	0.784	0.671	0.711	0.728	0.746	0.289	0.786 (0.779-0.794)
	15	0.779	0.677	0.714	0.729	0.745	0.286	0.785 (0.777-0.793)
	20	0.784	0.674	0.714	0.730	0.747	0.286	0.785 (0.777-0.793)
Logit-Boost	5	0.745	0.855	0.784	0.810	0.764	0.216	0.765 (0.755-0.774)
	10	0.746	0.855	0.784	0.810	0.764	0.216	0.765 (0.755-0.775)
	15	0.746	0.855	0.784	0.810	0.764	0.216	0.765 (0.755-0.774)
	20	0.628	0.911	0.787	0.814	0.698	0.213	0.715 (0.695-0.737)
Gradient Boosting	5	0.762	0.697	0.723	0.730	0.742	0.277	0.786 (0.778-0.794)
	10	0.770	0.684	0.716	0.728	0.742	0.284	0.784 (0.776-0.792)
	15	0.770	0.686	0.717	0.729	0.743	0.283	0.786 (0.777-0.794)
	20	0.774	0.682	0.716	0.729	0.744	0.284	0.785 (0.776-0.793)
LightGBM	5	0.791	0.661	0.707	0.727	0.747	0.292	0.800(0.792-0.807)
	10	0.790	0.661	0.707	0.727	0.746	0.293	0.799 (0.791-0.806)
	15	0.790	0.662	0.707	0.727	0.746	0.293	0.799 (0.792-0.807)
	20	0.791	0.661	0.707	0.727	0.747	0.293	0.800 (0.792-0.807)

**Fig 3:**
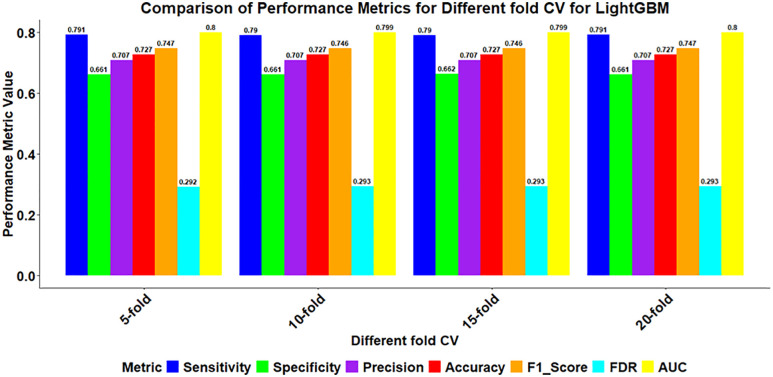
Comparison of performance metrics for different folds of CV for LightGBM.

Fig 3 presents the performance of the LightGBM model under four cross-validation schemes (5-, 10-, 15-, and 20-fold). With sensitivity ranging from 0.790 to 0.791, specificity from 0.661 to 0.662, precision fixed at 0.707, accuracy at 0.727, and F1-score between 0.746 and 0.747, the performance measures are remarkably stable throughout all validation folds. Similarly, the false discovery rate (FDR) stays constant at 0.292–0.293, but the AUC fluctuates just slightly between 0.799 and 0.800. These results show that the predictive performance of LightGBM is consistent across various cross-validation techniques, indicating that adding more folds to this dataset beyond five yields minimal incremental benefit.

**Fig 4:**
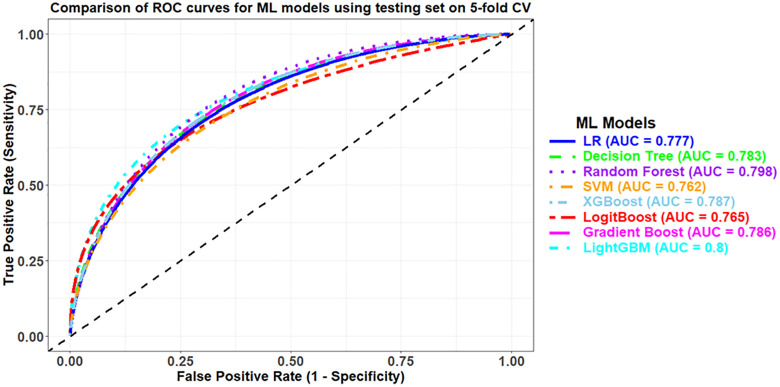
Comparison of ROC curves for eight ML models using the testing set on 5-fold CV.

**Fig 5:**
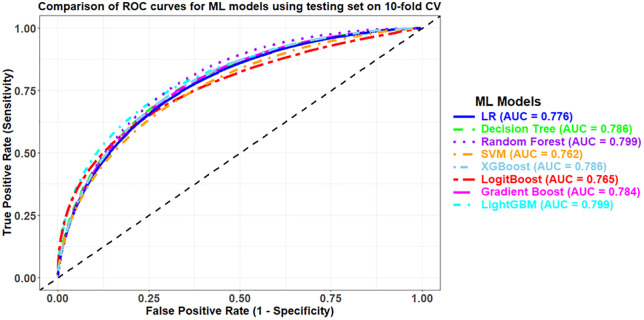
Comparison of ROC curves for eight ML models using the testing set on 10-fold CV.

**Fig 6:**
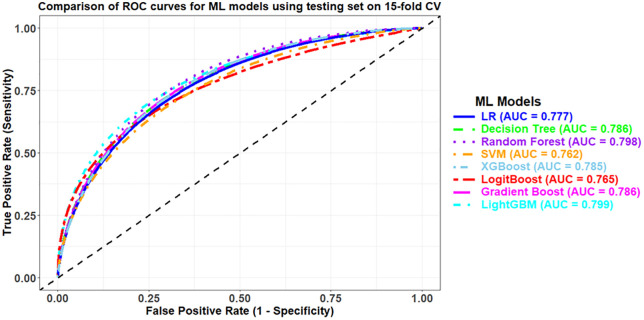
Comparison of ROC curves for eight ML models using the testing set on 15-fold CV.

**Fig 7:**
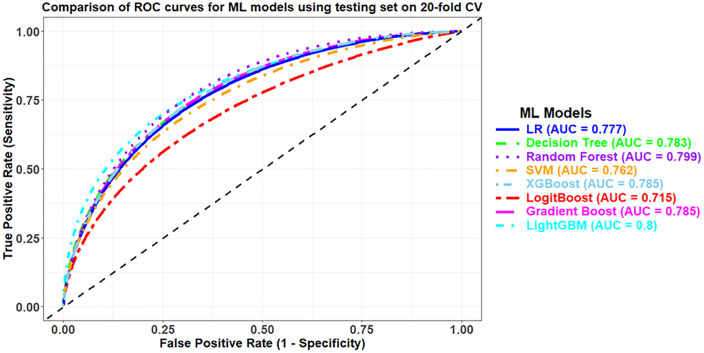
Comparison of ROC curves for eight ML models using the testing set on 20-fold CV.

All models’ results remain quite consistent across 5-, 10-, 15-, and 20-fold cross-validation, as indicated by the area under the receiver operating characteristic curve (AUC) in Fig 4,Fig 5,Fig 6,Fig 7 demonstrating steady discriminative performance. With consistently high AUC values across all folds (0.784–0.788), LightGBM outperforms the other models in overall discriminative ability between positive and negative cases. AUC values for Random Forest and XGBoost range from 0.785 to 0.787, indicating good but marginally poorer discriminative power than LightGBM. The lower AUC of 0.724 for LogitBoost, despite its excellent accuracy and specificity, indicated weaker class separation. The classification performance of SVM, Logistic Regression, Decision Trees, and Gradient Boosting is fair, as seen by their moderate AUCs (0.761–0.775). The model’s performance remains constant regardless of fold size, as evidenced by stable AUC values across several cross-validation folds. Overall, LightGBM is the recommended model based on AUC, as it offers the most consistent and reliable separation between positive and negative instances.

### 3.7. SHAP-based feature importance and contribution for the LightGBM model

**Fig 8:**
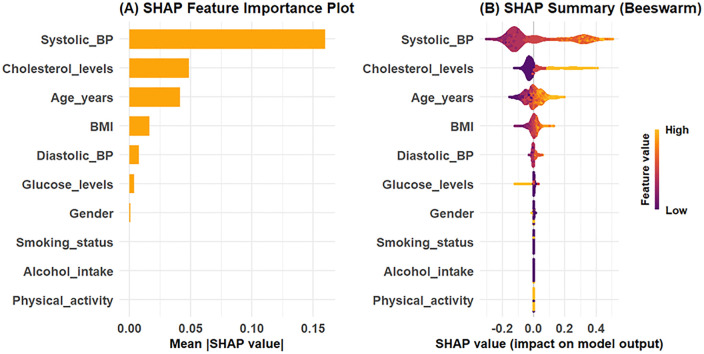
SHAP-based feature importance and contribution plot for LightGBM on 5-fold CV.

The relative contribution of each feature to the LightGBM model trained with 5-fold cross-validation is shown in Fig 8. With the highest relevance score (~0.65), systolic blood pressure is identified as the most significant predictor of cardiovascular disease, underscoring its crucial role in risk assessment. The next most significant characteristics, age and cholesterol, have a small impact on the model’s ability to predict outcomes. The minimal contributions of other clinical and lifestyle factors, such as BMI, diastolic blood pressure, physical activity, glucose levels, smoking status, gender, and alcohol consumption, indicate that the LightGBM model primarily relies on significant cardiovascular risk indicators for accurate prediction. This ranking aligns with accepted clinical knowledge, which states that the three main risk factors for cardiovascular events are elevated cholesterol levels, advanced age, and elevated systolic blood pressure. Feature importance reflects the predictive contribution of a feature within the model, not its causal relationships. Clinical causality requires randomized trials or alternative designs.

## 4. Discussion

This study uses the publicly accessible Kaggle dataset to perform a thorough comparative analysis of eight machine learning algorithms for cardiovascular disease (CVD) prediction. This work offers several significant scientific and practical advancements, including identifying LightGBM as the top-performing model for cardiovascular disease (CVD) prediction in 5-fold cross-validation. To identify reliable risk factors for CVD, a comparison of four advanced feature selection techniques (Boruta, RRF, RFE, and LASSO) reveals the consistency and stability of predictor importance. Second, a comparison of several cross-validation techniques indicates that 5-fold cross-validation offers a practical evaluation strategy, providing reliable performance at reduced computational cost. Third, the clinical relevance and interpretability of machine learning-based CVD prediction are enhanced by SHAP-based interpretability analysis of the best-performing model, which increases transparency by quantifying the contribution of individual predictors.

The dataset includes adults aged 39–64; the mean age is 52.89 years; there is substantial variation in body measurements; and there are significant differences in clinical indicators between groups with and without CVD. According to descriptive analysis, people with CVD are often older, heavier, and have higher BMI, systolic and diastolic blood pressures, cholesterol, and glucose levels than people without CVD. These results align with well-established clinical evidence that cardiovascular risk is associated with age, obesity, hypertension, and dyslipidemia. CVD cases are more likely to have behavioral variables, including physical inactivity, even when rates of alcohol and smoking are comparable among groups. Important variables, such as blood pressure and BMI show considerable deviations from normality, as indicated by normality tests, suggesting that robust machine learning techniques and nonparametric analyses are suitable for categorization.

Nonparametric comparisons highlight the potential of these characteristics for risk prediction, further confirming statistically significant differences between CVD and non-CVD groups across demographic, clinical, and lifestyle variables. Across 5-, 10-, 15-, and 20-fold cross-validation, LightGBM consistently produces the most balanced performance among the models studied, with the balanced F1-score (0.747), robust sensitivity (0.791), and AUC values ranging from 0.799 to 0.800. Although LogitBoost has the best overall accuracy (0.814) and specificity (0.911), its lower sensitivity (0.628) suggests that high-risk individuals may not have been fully detected. SVM demonstrates trade-offs between recognizing positive cases and maintaining overall classification balance, achieving the maximum sensitivity (~0.802). These results support earlier findings that complex nonlinear linkages and interactions among clinical variables are best captured by tree-based ensemble methods, particularly gradient-boosting frameworks such as LightGBM. The LightGBM model’s feature importance results indicate that systolic blood pressure is the most reliable indicator of cardiovascular illness. This finding is corroborated by earlier clinical and machine learning studies, which repeatedly found that blood pressure is one of the most important risk factors for correctly classifying heart patients [4,6,9,11].

In automated diagnostic systems, elevated systolic pressure is a key indicator, as it reflects increased arterial stiffness and vascular strain. Our analysis has found that age is the second most significant factor. In previous studies using hybrid and ensemble learning methodologies, it has been widely highlighted that cardiovascular vulnerability increases gradually with age [4,8]. Age increases the predictive power of algorithmic modeling by capturing cumulative exposure to metabolic stress, degenerative vascular alterations, and long-term lifestyle effects. The next most important factor was cholesterol levels. Lipid abnormalities are known to contribute to atherosclerosis and coronary problems, and comparative machine learning research has revealed that cholesterol-related variables are equally important [6,9]. Numerous studies have noted that adding cholesterol significantly enhances the stability and discrimination of the model. Body mass index also shows significant predictive power. It is well recognized that signs of obesity interact with diabetes, hypertension, and dyslipidemia to increase the risk of cardiovascular disease overall. Anthropometric parameters greatly improve detection accuracy when integrated into intelligent prediction systems, according to previous studies [7,11]. Although it is not a direct clinical factor, it can be a sign of underlying socioeconomic, developmental, or genetic issues.

Numerous studies on predictive modeling have observed similar secondary effects from these demographic traits [8]. Systolic blood pressure makes a larger contribution than diastolic blood pressure, consistent with earlier research showing that systolic measures provide better predictive power in machine learning frameworks [4,9]. When more powerful hemodynamic factors are present, the glucose level shows comparatively less significance. Previous research indicates that diabetes-related data may share variation with blood pressure and obesity, thereby lowering its independent ranking in multivariable algorithms [6]. Lifestyle factors like alcohol consumption, smoking status, and physical activity are placed close to the bottom of the significance scale. Although these are recognized contributors to cardiovascular morbidity, several studies have shown that, once clinical indicators are incorporated into the model, their predictive power often becomes indirect [4,7,9]. Furthermore, their quantifiable impact can be undermined by possible reporting bias. Gender is not very important on its own. Simultaneous modeling of main metabolic and physiological factors has been shown to reduce sex-based effects similarly [8,11]. According to our analysis, objective medical measurements outweigh behavioral markers, which aligns with the body of research on intelligent cardiovascular prediction systems. It has been consistently shown through comparative analyses of various algorithms that ensemble and boosting methods prioritize the strongest predictors of classification performance, including age, lipid profile, blood pressure, and obesity markers [4]- [11].

The results of this study imply that predictive models based on machine learning might be useful in assisting with the assessment of cardiovascular disease risk. In addition to helping doctors prioritize preventative measures or more clinical examination, these models may help to identify individuals at elevated risk. Even though the suggested models show potential for predicting cardiovascular disease, careful consideration should be taken when interpreting their application in clinical practice, mobile health platforms, or electronic health record systems. Before practical implementation, additional external validation, calibration assessment, prospective testing, and evaluation within actual clinical workflows are required.

### Strengths and limitations

This study shows consistent results across several cross-validation strategies by comparing eight machine learning models within a standardized framework. The robustness of predictor identification is increased through the application of several feature selection strategies. Furthermore, the use of common, accessible clinical variables enhances the practicality of application. The combination of predictability and interpretability strengthens the therapeutic value of the findings. The generalizability of the analysis to other populations may be limited because it is based on a single public dataset. Time-based risk assessment and causal interpretation are not possible due to the cross-sectional design. Certain lifestyle factors can be subject to reporting bias, which can lessen their apparent impact. The lack of comprehensive biomarker or imaging data may also have limited further gains in predictive accuracy. A methodological limitation of this study is the inclusion of higher-fold cross-validation schemes (15- and 20-fold), which may increase computational cost and training set overlap. However, compared with 5-fold cross-validation, only slight performance differences are observed, suggesting that lower-fold methods may offer a more reliable and computationally efficient evaluation approach for large-scale cardiovascular disease datasets. Despite achieving the best predictive performance, LightGBM’s observed accuracy (72.7%) still indicates a significant misclassification rate, which could limit its application for independent clinical decision-making. Thus, rather than taking on the role of clinical judgment, the suggested models should be considered decision-support tools to aid clinical assessment. Future research may enhance performance by adding more biomarkers and more comprehensive clinical data. Another limitation of this study is the absence of external validation using an independent dataset, which may limit the generalizability of the findings across different populations and clinical settings. Therefore, further validation in a variety of cohorts is required prior to clinical deployment. Furthermore, decision curve analysis, calibration assessment, and clinical utility evaluation are beyond the scope of this study but are essential prior to clinical implementation.

## 5. Conclusion and future research

This study uses a publicly available dataset to compare eight machine learning algorithms for predicting cardiovascular disease. The results show that ensemble boosting techniques outperform conventional techniques in achieving balanced, reliable classification. Among the evaluated models, LightGBM demonstrates modestly better, more balanced predictive performance, achieving comparatively higher F1-score and AUC values across multiple cross-validation schemes. The most significant cardiovascular risk factors are systolic blood pressure, cholesterol levels, and age as further supported by SHAP-based feature importance technique. The manuscript provides modest but methodologically incremental knowledge, primarily in demonstrating feature importance instability across selection methods, establishing an optimal cross-validation scheme for this dataset, and identifying the CV-dependent behaviors of the selected models. However, it does not contribute new knowledge to cardiovascular pathology and relies on well-established risk factors. The primary value lies in the methodological rigor applied to algorithm comparison and validation, rather than in novel clinical or biological discovery.

Even though the results demonstrate the promise of machine learning techniques as decision-support tools for cardiovascular risk prediction, additional clinical utility assessments and external validation across diverse populations are required before wider clinical deployment. By including additional data sources, such as wearable sensor outputs, lab biomarkers, imaging data, or genetic information, future studies may enhance predictive performance. To improve predicted performance while preserving interpretability, explainable AI, deep learning, and hybrid ensemble techniques may be investigated.

## Supporting information

S1 Table Number of outliers in each quantitative variables. (DOCX)

S2 Table One-sample Kolmogorov-Smirnov (K-S) test. (DOCX)

S3 Table Statistical comparison of ROC-AUC between LightGBM and Logistic Regression using bootstrap and DeLong tests. (DOCX)

S4 Table Sensitivity analysis of LightGBM performance with and without outlier removal under different cross-validation schemes. (DOCX)
