# Supplementary material for: Comparative analysis of machine learning techniques for cardiovascular disease prediction
Source: PLoS One. 2026 Aug 18;21(8):e0356170. doi: 10.1371/journal.pone.0356170 (PMC13485063; doi:10.1371/journal.pone.0356170)
Supplement: Supplementary file 1 [file pone.0356170.s001.docx]

**Table S1:** Number of outliers in each quantitative variable

| **Variables name** | **Number of outliers** |
| --- | --- |
| Age (in days) | 4 |
| Height | 502 |
| Weight | 1711 |
| Systolic BP | 648 |
| Diastolic BP | 1637 |
| Age years | 4 |
| BMI | 1988 |
| **Total** | **6,494** |
