# Supplementary material for: Comparative analysis of machine learning techniques for cardiovascular disease prediction
Source: PLoS One. 2026 Aug 18;21(8):e0356170. doi: 10.1371/journal.pone.0356170 (PMC13485063; doi:10.1371/journal.pone.0356170)
Supplement: Supplementary file 2 [file pone.0356170.s002.docx]

**Table S2:** One-sample Kolmogorov-Smirnov (K-S) test

| Variables | Test statistic (D-value) | P-value |
| --- | --- | --- |
| Age (days) | 0.061 | <0.001 |
| Height | 0.052 | <0.001 |
| Weight | 0.075 | <0.001 |
| Systolic BP | 0.267 | <0.001 |
| Diastolic BP | 0.304 | <0.001 |
| Age (years) | 0.072 | <0.001 |
| BMI | 0.074 | <0.001 |
