# Supplementary material for: Comparative analysis of machine learning techniques for cardiovascular disease prediction
Source: PLoS One. 2026 Aug 18;21(8):e0356170. doi: 10.1371/journal.pone.0356170 (PMC13485063; doi:10.1371/journal.pone.0356170)
Supplement: Supplementary file 3 [file pone.0356170.s003.docx]

**Table S3:** Statistical comparison of ROC-AUC between LightGBM and Logistic Regression using bootstrap and DeLong tests

| Comparison | AUC difference | Test method | Test statistic | P-value |
| --- | --- | --- | --- | --- |
| LightGBM vs Logistic Regression | 0.0044 | Bootstrap test (1,000 resamples) | D = 0.777 | 0.437 |
| LightGBM vs Logistic Regression | 0.0044 | DeLong test | D = 0.773 | 0.440 |
