# Supplementary material for: Comparative analysis of machine learning techniques for cardiovascular disease prediction
Source: PLoS One. 2026 Aug 18;21(8):e0356170. doi: 10.1371/journal.pone.0356170 (PMC13485063; doi:10.1371/journal.pone.0356170)
Supplement: Supplementary file 4 [file pone.0356170.s004.docx]

**Table S4:** Sensitivity analysis of LightGBM performance with and without outlier removal under different cross-validation schemes

| Model | CV (folds) | Performance metrics | | | | | | |
| --- | --- | --- | --- | --- | --- | --- | --- | --- |
|  |  | Sensi-  tivity | Speci-ficity | Preci-sion | Accu-racy | F1- score | FDR | AUC (95% CI) |
| LightGBM(Without Outliers) | 5 | 0.791 | 0.661 | 0.707 | 0.727 | 0.747 | 0.292 | 0.800 (0.792-0.807) |
|  | 10 | 0.790 | 0.661 | 0.707 | 0.727 | 0.746 | 0.293 | 0.799 (0.791-0.806) |
|  | 15 | 0.790 | 0.662 | 0.707 | 0.727 | 0.746 | 0.293 | 0.799 (0.792-0.807) |
|  | 20 | 0.791 | 0.661 | 0.707 | 0.727 | 0.747 | 0.293 | 0.800 (0.792-0.807) |
|  |  |  |  |  |  |  |  |  |
| LightGBM  (With Outliers) | 5 | 0.787 | 0.684 | 0.719 | 0.736 | 0.751 | 0.281 | 0.794 (0.786-0.801) |
|  | 10 | 0.789 | 0.680 | 0.716 | 0.735 | 0.751 | 0.284 | 0.806 (0.798-0.813) |
|  | 15 | 0.789 | 0.680 | 0.716 | 0.735 | 0.751 | 0.284 | 0.806 (0.798-0.813) |
|  | 20 | 0.786 | 0.684 | 0.718 | 0.736 | 0.751 | 0.282 | 0.796 (0.788-0.803) |
